# Cerebrospinal Fluid Pharmacology: An Improved Pharmacology Approach for Chinese Herbal Medicine Research

**DOI:** 10.1155/2013/674305

**Published:** 2013-12-16

**Authors:** Yan-qing Wu, Ying-wu Zhou, Xiu-de Qin, Sheng-yu Hua, Yu-lian Zhang, Li-yuan Kang

**Affiliations:** ^1^Institute of Traditional Chinese Medicine, Tianjin University of Traditional Chinese Medicine, Tianjin 300193, China; ^2^The Second Hospital Affiliated to Tianjin University of Traditional Chinese Medicine, Tianjin 300150, China; ^3^The Gu Lou Hospital of Traditional Chinese Medicine of Beijing, Beijing 100009, China

## Abstract

Despite many successful applications of Chinese herbal medicine (CHM) in the treatment and prevention of neurological diseases (ND), the fully scientific understanding of CHM's action mechanisms had been hampered for lack of appropriate methods to explore the combinatorial rules, the synergistic mechanisms, and the molecular basis of CHM. As an improved pharmacology approach, cerebrospinal fluid pharmacology (CSFP), based on the fact that cerebrospinal fluid plays an important role in the health maintenance of specific survival environment for neurons and glial cells, has been constructed and applied to CHM research for treating ND. In the present review, the concept and advantages of CSFP are briefly introduced. The approaches and key technologies of CSFP in CHM research are also collated and analyzed. Furthermore, the developing tendency of CSFP is summarized, and its framework in CHM research is also proposed. In summary, CSFP provides a new strategy not only to eliminate some barriers of CHM research for treating ND, but also to broaden the pharmacology research for bridging the gap between CHM and modern medicine. Moreover, the advancements in CSFP will bring about a conceptual move in active ingredients discovery of CHM and make a significant contribution to CHM modernization and globalization.

## 1. Introduction

Many neurological diseases (ND) are caused or exacerbated by disparate physiological, pathological, environmental, and lifestyle factors. It is obvious that the hypothesis to search for highly selective single-target drugs interacting with a well-defined molecular target is meeting the challenges owing to the complex etiology and pathogenesis of ND. As the leading cause of disability, ND has attracted unprecedented attention from medical researchers around the world, and different types of drugs and medications are used for treating ND, such as intensive medication, rehabilitation therapy, and traditional Chinese medicine (TCM). TCM has been reevaluated and considered as one of the most important complementary or alternative medicine in most western countries and has been increasingly accepted worldwide [1]. Pharmaceutical companies have increasingly shifted their attention toward TCM for novel lead compounds, and increasing efforts have been devoted to study TCM, from which a large number of bioactive compounds have been isolated and studied [2–6]. Chinese herbal medicine (CHM), with the concept of multitarget or multicomponent therapy, has accumulated rich experience in treating ND and a large number of medical records [7–11] and drug design studies [12–16] published in international journals. These records not only enable people to understand the mechanisms of CHM with a new perspective, but also provide a modern theoretical basis for clinical applications. As an important potential source for the discovery of bioactive molecules with therapeutic effects, CHM is gaining increased attention for designing multitargets drugs, and domestic and foreign scholars have been devoted to elucidating the efficacy mechanisms and the material basis of CHM which belong to natural products and have already been proved to possess clear therapeutic action on public health in the clinical setting [17–26]. However, the fully scientific understanding of CHM's action mechanisms has been hampered by the lack of appropriate methods that can explore the combinatorial rules, the synergistic pharmacological mechanisms, and the complex nature of CHM at the cellular and molecular levels. Thus how to make the cell biology technique in vitro coincides with the characteristics of CHM treatment is one of the major issues that need to be addressed.

Serum pharmacology is an external testing method to explore the mechanism of CHM at the cellular and molecular levels. However, owing to the presence of blood-brain barrier and the specific survival environment of neurons and glial cells in central nervous system (CNS), serum pharmacology has some limitations in CHM researches for treating ND. As a methodology and technology, cerebrospinal fluid pharmacology (CSFP) is an emerging pharmacological method mainly to the research of drugs for treating ND [27]. Since CSFP imitated the survival microenvironment of neurons and glial cells in CNS, the application of this method could improve the credibility of CHM pharmacology by eliminating the interference caused by the physical and chemical properties of the other solvent itself containing CHM. In addition, CSFP, as an improved pharmacology method in vitro, not only provides a new scientific research method to the effectiveness evaluation system of CHM, but also brought new ideas in studying the material basis and the efficacy mechanism of CHM. Moreover, this principle obviously coincides with the synergistic effects of multiple compounds and herbal formula, which are mainly based on the integrative and holistic ways.

In this present review, the concept and significance of CSFP are briefly introduced. Its application and potential role in CHM research are also collated and analyzed. Furthermore, the limitations and problems of CSFP are also discussed, and its framework for bridging the gap between CHM and modern medicine is proposed.

## 2. Concept and Approaches of CSFP in CHM Research 

Pharmacology has provided fresh insight into the drug discovery. However, addressing the scientific suspense on the material basis and the efficacy mechanism of CHM is urgent in order to develop a pharmacological methodology based on the characteristics of CHM. Yan et al. [28] proposed that the methodology in CHM research must follow three principles of these combinations, that is, the combination of systems theory and reductionism, the combination of macroeconomic research and microscopic studies, and the combination of analysis in vivo and analysis in vitro, which pointed out the overall direction and principles of CHM research.

In order to investigate the action mechanism of CHM at the cellular level in vitro, serum pharmacology was adopted in the pharmacological investigation and the drug screening of the CHM [29]. serum pharmacology provides profitable approach to address some scientific suspense of CHM studies in vitro. However, along with the deepening of CHM research, there are some obvious flaws when using serum pharmacology to study CHM for treating ND. Therefore, the pharmacological method reflecting the material basis and mechanism of CHM for treating ND is really becoming the focus in modern CHM research. In 1999, to explore an effective method by which the pharmacological effects of CHM for treating ND could be correctly investigated, Mei et al. [30], inspired by the idea of serum pharmacology and based on the innovation and improvement of early methodology in the course of CHM research, created a novel concept of CSFP, which built on the fundamental concept that cerebrospinal fluid (CSF) is the immediate survival environment of neurons and glial cells in CNS.

The CSFP was informally established based on the study of Qingnao Yizhi prescription's effect on neurons damaged by glutamate. CSF and serum were, respectively, taken in 1–1.5 h after administration of CHM and added into the culture medium of astrocytes. After 48 h, a certain amount of the incubated medium was transferred into the neuron culture medium. The serum, but not the CSF, showed a certain degree of cytotoxicity on the astrocytes. The astrocytes medium stimulated by the CSF containing Qingnao Yizhi prescription could promote the neuron axon growth. The experiment verified the superiority of CSF to serum and the validity of the CSFP to evaluate the action mechanism of CHM in vitro, thus indicating that the CSFP is suitable CHM study [27]. Briefly, the CSFP is using CSF extracted from animals after administration of drugs to explore the material basis and the efficacy mechanism in vitro experiment. CSFP coincides with the fundamental characteristics of CHM in neurological therapeutic areas that many active substances are the metabolites or secreted products passed through blood-brain barrier to the CSF. It affords a rewarding assistance to the authenticity and reliability of the experimental results at a higher efficiency, and to improve the potency for drug discovery.

## 3. Advantages of CSFP in CHM Research

CSFP provided a new method to broaden the pharmacology research of CHM. This method has the following advantages. Firstly and most obviously, the physical and chemical properties of CSF are very similar to the survival environment of neurons and glial cells in CNS. CSFP could eliminate the interference caused by the physicochemical properties of the other solvent itself containing CHM, thus improving the credibility of CHM pharmacology. Secondly, metabolic inactivation, without being absorbed into the body, indirectly exert their therapeutic effects through signaling cascades such as 2, 3 messenger, which are the major pathways transformed into active ingredients from chemical ingredients of CHM. Thus makes the research for material basis and action mechanisms of CHM very difficult [31]. Both the active ingredients produced by a series of vivo biotransformation of CHM and the endogenous active ingredients produced by the body under the action of CHM are the pharmaceutical ingredients in CSF containing CHM. CSFP not only simulates the interaction between drugs and the body, but also precludes various confounding factors in these crude extracts of CHM, thus improving scientific authenticity and feasibility in the research to clarify the efficacy material basis and action mechanisms of CHM for treating ND in vitro experiments. Thirdly, these factors affecting the experimental results in vitro, such as the toxic effects of the serum itself and the various questions on whether and which active ingredients of CHM passed through the blood brain barrier could be eliminated by using CSFP to explore the efficacy of CHM for treating ND, thus contributing to confirm the active components and the bioactive ingredients based on the efficacy of CHM for treating ND. Finally, using CSFP to explore the efficacy relationship and the dynamic changes of the active ingredients in CSF contributs to clarify the essence of CHM compatibility, thus providing a new method to promote the basic theory research of CHM.

## 4. Key Technologies of CSFP in CHM Research

As an emerging pharmacology used mainly to evaluate the material basis and action mechanisms of CHM in treatment of ND, the experimental conditions for CSFP are very immature and needed further improvement.

### 4.1. Selection of Animals Used to Extract CSF

The first key of CSFP is how to obtain effective CSF containing CHM. Theoretically, the animal used to extract CSF and the animal used to isolated cells should belong to the same genus, thus narrowing the differences in the physical, chemical, and biological characteristics of the two kinds of CSF and reducing the immune response owing to the species differences. Ma and Tian [32] explored the effect of the CSF containing Liuwei Dihuang pills (LDP) components extracted from different species (human, rabbit, and rat) on PC12 cell injured by A*β*_1−40_. Surprisingly, there is no evident difference of the cell viability among the CSF containing LDP extracted from human group, the CSF containing LDP extracted from rabbit group, and the CSF containing LDP extracted from rat group. Furthermore, further studies on selection of animals in CSFP are needed to be validated.

### 4.2. Optimal Dose and Timing of Administration to Animals

In order to obtain the high-quality CSF containing drug, determining the timing of administration to animals should depend on the half-life of drug. CSF was taken from rabbits mainly in the period of 1–1.5 h [27], 1 d [33], 3 d [34–37], 3.5 d [38, 39], 7 d [40], and 15 d [41] after consecutive administration of CHM in currently CSFP studies. CSF was taken from rats after consecutive administration of CHM for 3 d and 3.5 d. CSF was taken from beagle dogs after administration of CHM for 2 months [42]. Owing to the complex composition of CHM, the time-effect relationship of CHM was probably used to determine the optimal time period. Studies demonstrated that CSF extracted from rat after administering Danggui Shaoyao powder or Nimodipine repetitiously (2  times/d × 3.5 d) exerted higher protective efficacy on PC12 cell than that after single administration of Danggui Shaoyao powder or Nimodipine [43, 44]. It is proposed that the CSF containing drugs should be extracted from animals after administerting drugs repetitiously.

In order to obtain CSF containing higher concentration drugs, most of the experimental researches intend to increase the dose to administration. It is noted that simply increasing the dose of administration to the animals is not necessarily a benefit to absorption, distribution, and metabolism of CHM, and thus increasing the CHM components in CSF. Conversely, CSF containing very high concentrations of drugs inhibited the vitality of neurons [45]. Therefore, in order to make the test conditions closer to the environment of the drug effect in the body, the optimal dose administrated to related animals should be converted according to the standard conversion rules of the adult dosage [34, 46].

### 4.3. Acquisition Time of CSF Containing Drugs

Since the different process of absorption, distribution, and metabolism are caused by various features of CHM and its multiple pharmaceutical formulations, thus, the active ingredients and thier amounts in CSF containing drugs are different to collect at different times. To verify the optimal acquisition time of CSF containing Tongsaimai tiny pill extracted from rats, Zhang et al. [47] explored the efficacy of CSF containing Tongsaimai tiny pill, collected at range from 0.5 to 3 hours after the last administration, following consecutively administration for 3 days on injured PC12 cells. The results demonstrated that the optimal acquisition time of CSF containing Tongsaimai tiny pill is at 2 hours after the last administration. Cui et al.'s experiment demonstrated that the optimal acquisition time of CSF containing Qingxin Kaiqiao decoction is also at 2 hours after the last administration [48]. In addition, Zhang et al.'s studies showed that 1.5 hour after the last administration is the optimal acquisition time of CSF containing Danggui Shaoyao powder [43].

### 4.4. Acquisition Methods of CSF Containing Drugs

Precise location for puncture is the key point of drawing CSF from experimental animals. Fu et al. [49] observed anatomic structure of cerebellomedullary cistern in rabbit and rat to decide the optimal percutaneous puncture position and direction. The district of cerebral dura mater covering over the cerebellomedullary cistern was thin, soft, and frangible to be pierced. The district projected on the skin of neck is in between external occipital protuberance and first cervical vertebra. The optimal percutaneous puncture positions for rabbit and rat were about 1.0–1.2 cm and 0.6–0.7 cm below the center of external occipital protuberance, respectively, when animals' head bended to thorax. The pinhead paralleled the parietal bone to pierce the skin and cerebral dura mater, and then reached cerebellomedullary cistern. The percutaneous puncture to this direction can effectively avoid injuring the medulla oblongata and blood vessels. Further studies [50, 51] also demonstrated that the method to collect CSF from foramen magnum is visual and convenient to operate, causing less injury and complications and possessing a high rate of success. In addition, the collection volume and the pass rate of using microinjector via spinal dura mater puncture under direct vision were much higher than those of the cerebellomedullary cistern via percutaneous puncture and the gashed spinal dura mater under direct vision (*P* < 0.01) [52].

### 4.5. Preservation, Processing, and Additive Amount of CSF

The impact probably caused by preservation, processing, and additive amount of CSF containing drugs cannot be ignored at some main approaches in the drug development process. To establish the standard conditions in preservation, processing, and additive amount of CSF is very important to the CSFP method. How to process CSF is the first issue to consider. Surprisingly, The study of Zhang et al. [44] illustrated that there was no obvious statistical difference among the raw CSF containing Nimodipine and the CSF containing Nimodipine treated by heat (56°C), ethanol, or acetone, through the experiment to explore the protective effect of CSF containing Nimodipine on injured PC12 cells induced by the hydrogen peroxide under different conditions. Meanwhile, there was also no significant statistical difference of the protective effects on injured PC12 cells among the raw CSF containing optimized Danggui Shaoyao powder and the CSF containing Danggui Shaoyao powder treated by heat (56°C), ethanol, or acetone, through the experiment to explore the protective effect of CSF containing optimized Danggui Shaoyao powder on PC12 cells injured by the hydrogen peroxide under different conditions [43]. Thence, it is not necessary for the processing of CSF containing drugs after extracting from animals.

Preservation of CSF containing drugs is the key point of ensuring the authenticity with the experiment results. The study of Zhang et al. [44] demonstrated that the protective rates of CSF containing drugs preserved for 1 d, 15 d, and 30 d at −20°C cryopreservation were, respectively, 65.43%, 50.18% and 24.86% in the experiment to explore the protective effect of CSF containing drugs on PC12 cells injured by hydrogen peroxide. It is no doubt that the protective rates declined with the prolonging of the preserved time. It is suggested that the CSF containing drugs at −20°C cryopreservation are preserved no more than 30 days. In addition, the general added amount of CSF containing drugs is 10% or 20% of the medium in current studies [43, 44].

In summary, in order to make the test conditions closer to the environment of the drug effect in the body, it is suggested that the CSF containing drug may be prepared with 10% or 20% CSF without treating after being extracted from animals and preserved at −20°C no more than 30 days.

## 5. Application of CSFP in CHM Research

**Figure 1:**
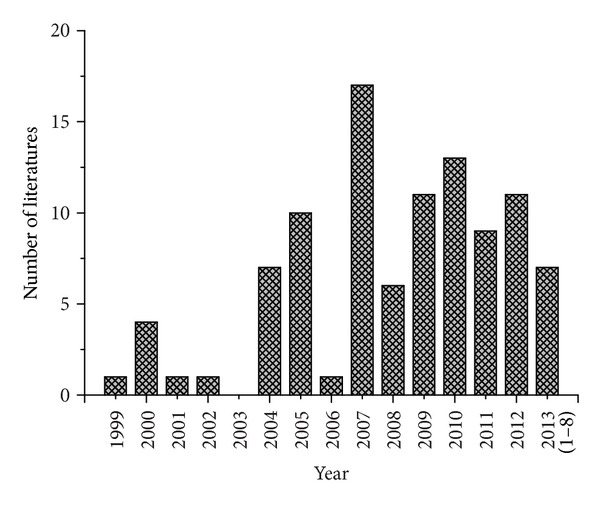
Developing tendency of CSF pharmacology study for CHM research. The publications of CSF pharmacology for CHM research in PubMed and CNKI databases from 1999 to August 2013. All results were screened in manual way.

CHM, the ancient traditional treatment methodology popular in China and surrounding areas, has been recognized as a potential important pharmaceutical area of TCM and holds promise for preventing diseases in a holistic way [53]. In a very long period of clinical practice, it is known for its effectiveness and beneficial contribution to public health and disease control. However, the modern pharmacological mechanisms of CHM have not been fully established [54]. With increasing knowledge of the genes and molecular interactions, the researchers intend to adopt some pharmacology for CHM research and development. Up till now, CSFP in CHM research has been frequently reported.  Figure 1 shows the developing tendency of CSFP studies from the data available in PubMed and China National Knowledge Infrastructure (CNKI) databases from 1999 to August 2013. The applications of CSFP, focused on the studies of CHM from classic and commonly used prescriptions for treating ND, were systematically summarized to demonstrate the significant value.

### 5.1. Verify the Efficacy and Action Mechanism of CHM for Treating ND

To explore the efficacy and action mechanism of Qingnao Yizhi formula (QYF) for treating vascular dementia and multiinfarct dementia, Mei et al. [55, 56] utilized CSFP to investigate the effect of CSF-QYF on the cultured rabbit neurons injured by glutamate. CSF was taken at 1–1.5 h after administration of CHM and added into the culture medium of astrocytes. After 48 h, the incubated medium was transferred into the neuron culture medium according to a certain proportion. The results demonstrated that QYF could increase the survival rate of injured neuron, decrease LDH content in the culture medium, and increase the neuron survival activity in the culture medium. Meanwhile, it had no effect on the proliferation of astrogliocytes. These results showed that QYF can protect neurons from glutamate-induced injury by stimulating the secretion of neurotrophic factors from the astrogliocytes.

To investigate the protective effects of Jiawei Wuzi Yanzong formula (JWYF) on hippocampal neurons injured by beta-amyloid, an experimental model of Alzheimer's disease in vitro, Zeng et al. [57] used CSFP method to investigate the effect of CSF extracted from SD rats, respectively, fed with various components of JWYF (total formula, total flavonoids, or total polysaccharides) on beta-amyloid protein-induced neurons. CSF containing JWYF showed significant neuroprotective effect, and the protection of CSF containing total flavonoids or total polysaccharides was significantly greater than that of CSF containing total formula. The results showed that some flavonoids and polysaccharide components in JWYF can pass through the blood-brain barrier and protect neurons from beta-amyloid protein-induced neuron injury to some extent.

To investigate the protective effects of the CSF with ancient prescriptions on PC12 cells injured by *β*-amyloid protein (A*β*_25–35_), an experimental model of Alzheimer's disease in vitro, several studies manifested that CSF containing Dihuang decoction (CSF-DHD) can effectively reduce calcium ion internal flowing, reduce the protein expression of c-jun, c-fos, bax, caspase-3, and APP, enhance the expression of bcl-2, and improve the survival condition and the vitality of PC12 cells, following injury by A*β*_25–35_, thus indicating that CSF-DHD has a protective effect on injured PC12 cell induced by A*β*_25–35_ [58–62]. In addition, CSF-DHD can effectively decrease the releasing of LDH and increase the live activity of cultured hippocampal neurons injured by A*β*_25–35_ [63].

PC12 cell induced by MPP+, an experimental model of Parkinson disease in vitro, was utilized to investigate the mechanism of ancient prescriptions on Parkinson disease. Studies showed that CSF containing Zhengan Xifeng decoction (CSF-ZGXFD) could remarkably restrain the expression of caspase-3 mRNA, Cyt c protein, phospho c-jun protein, mRNA and protein of Bax, and mRNA and protein of Bcl-2 and significantly upregulate mRNA and protein expression of Bcl-2 in PC12 cell induced by MPP+, thus indicating that CSF-ZGXFD has protective effects on injured PC12 cell induced by MPP+ [64–66].

The injured PC12 cells, cell models of ischemic stroke induced by hydrogen peroxide, sodium dithionite and glutamic acid respectively, was used to clarify the mechanisms of CHM on ischemic stroke. Yan et al.'s [67] experiment showed that CSF containing Tongsaimai tiny pill has a protective effect on injured PC12 cell by inhibiting intracellular calcium overload, free radical oxidative damage, and glutamate excitotoxicity.

Astrogia CRL-2541 cell injured by the high ammonia, a cell model of hepatic encephalopathy (HE), was utilized to explore mechanism of Ruhuang pill on hepatic encephalopathy. Studies [67, 68] showed that CSF containing Ruhuang pill increased the vitality of CRL-2541 cells injured under high ammonia and upregulated the GFAP expression, indicating that Ruhuang pill could enter in BBB and protect the injured astroglia in high ammonia, which suggests that astroglia is one of the targets of Ruhuang pill for treating HE.

### 5.2. Clarify Effective Constituents of CHM

Effect of CHM on neurological disease is based on comprehension of effective constituents of CSF containing CHM, which utilizes the CSFP analysis to speculate and estimate effects of drugs. It focuses on the specific component comparison between CSF containing CHM and serum containing CHM and provides assistance for drug effective evaluation and research. To confirm the main effective constituents in Sinisan freeze-dry powder with the improving sedative-hypnotic function, Li et al. [69, 70] used CSFP to explore the main active constituents of Sinisan freeze-dry powder. Surprisingly, the study identified that there were no other constituents of Sinisan freeze-dry powder absorbed into blood except for the pentobarbital natrium. However, the endogenous substance in CSF containing Sinisan freeze-dry powder was increased obviously. Synephrine, paeoniflorin, saikosaponin C, and glycyrrhetinic acid could make the peak area of endogenous substance (5-hydroxytryptamine, 5-HT) in CSF larger than that in blank CSF. But all their effects were less than those of Sinisan freeze-dry powder. The increasing peak area of 5-HT in CSF containing the mixture (synephrine : paeoniflorin : saikosaponin C : glycyrrhetinic acid = 17 : 2 : 3 : 13) was 3.2 times as much as that of Sinisan freeze-dry powder, thus indicating that the active constituents (synephrine, paeoniflorin, saikosaponin C, and glycyrrhetinic acid) in Sinisan freeze-dry powder are confirmed and could improve the sedative function.

### 5.3. Determine the Therapeutic Time Window of CHM

The chronomedicine theory of TCM is an integral part of the treatment in CHM [71–73]. From a macroperspective, it reflects the rhythm of human physiological activities and organs activities to regulate body functional imbalances and disorders. CSFP could be used for determining the therapeutic time window of CHM to guide clinic treatment, which integrates the information of “heaven corresponding humans” and emphasizes the close relationship between the body's natural rhythms.

The injured neurons induced by thrombin and hypoxic-ischemic, a cell model of acute intracerebral hemorrhage (AICH) in vitro, were used to observe the therapeutic time window of AICH intervened by Beagle dog CSF containing DSQ-03 herbal medicine. Guo et al.'s experiment revealed that beagle dog's CSF containing DSQ-03 was able to inhibit the neural apoptosis; the therapeutic time window of AICH treated with DSQ-03 was at a time from 0 h to 24 h following intracerebral hemorrhage. Meanwhile, the study laid the foundation to further investigate the efficacy mechanism and to determine the clinical dose [74].

### 5.4. Verify Drug Association and Perfect Quality Standard in CHM Prescription

Monarch, minister, assistant, and guide in CHM prescription contain many principles of system theory, and the aim of coordination and cooperation in several kinds of CHM is to regulate body functional imbalances and disorders [54]. CSFP could be used to verify drug association and perfect quality standard of CHM prescription based on “the mechanisms of multicomponent efficiency in CHM” model. Shen et al. [75] used CSFP method to describe the properties of the effective constituents in Dachuanxiong prescription. The result revealed that senkyunolide I is the only ingredient found both in plasma and in CSF, thus indicating that senkyunolide I is the guide component to upstream of effective ingredients. Research on key ingredients of active components in Dachuanxiong prescription is favorable to the clarification of its active substance basis and perfection of quality standard. CSFP offered valuable information to identify the effective constituents and potential effect of known compounds in a complex system.

Due to limited space, only 4 kinds of literature reports related to CSFP containing CHM were provided, each of which was published by mainly Chinese scholars in recent years. Although the studies introduced in this paper are not thorough and systematic enough, there is still no doubt that the CSFP plays an effective role in studying the efficacy mechanism of CHM and solving the problems of administration in vitro experiment. Brief information of CSFP technologies and tools is shown in Table 1.

## 6. Discussion and Conclusion 

CHM has a unique theoretical system which belongs to the dialectical and philosophical medical system and mainly reflects the features of the experience-based medicine. Its clinical practice is based on the theory of the holistic concept and syndrome differentiation [76]. In terms of TCM theory, the pharmacological effect of CHM has the advantages of the overall adjustment, bidirectional regulation, and multiple prevention-treatment-repairing in physiological and pathological conditions. That is to say, CHM has multitarget actions, rather than single effect, which are completely different from the modern medical system [11]. Therefore, if the research ideas of TCM completely follow the research methods of the modern medicine such as the separation of the chemical composition and screening of the active ingredient which could be directly used in vitro experiment, there are some problems and flaws ignoring TCM characteristics of the multitarget synergistic overall effect in the methodology contrary to the basic theory of TCM, thus affecting the authenticity and reliability of the experimental results. Unclear material basis, unclear action mechanism, and low-level quality control are the main problems more or less existing in CHM preparations. The evaluation and validation of the safety and efficacy of CHM, CHM prescriptions, and treatment techniques are key points. Therefore, we should attach great importance to basic research on material basis and compatibility law of CHM combined with modern medical indexes at the cellular level. The biggest obstacles of CHM development are lack of appropriate methods to really explain the effective mechanisms and the scientific connotation of the CHM formulae.

Owing to the presence of blood-brain barrier and the specific survival environment of neurons and glial cells, it is no doubt that serum pharmacology is not suitable to evaluate the effects of CHM for treating ND. Fortunately, as a novel approach, CSFP imitated the survival microenvironment of neurons and glial cells to optimize the discovery of drug for treating ND. Since the CSFP was proposed, it is gradually becoming an important approach to explore the details of drug-target, material basis, and efficacy mechanism of multiple active components in CHM for treating ND. As seen in the above studies, CSFP was suitable to such studies, providing a robust basis and direction for basic research. In addition, CSFP can truly reflect pharmacological effect and increase the relevance of active ingredients and should be adopted in the pharmacological investigation and the drug screening of CHM. So it is thought that CSFP has an important scientific significance at the cellular level.

The application of CHM is inseparable from the guiding of CHM theory. It does not matter whether CHM could pass through BBB, it is likely to play a role in treating ND [77], thus also consistent with the theory that CHM has the multitarget actions of the overall adjustment. Yao et al.'s [60] experiment proved the authenticity of this theory.

We can draw a conclusion that all these roles may be the result of maintaining balance and harmony of the internal environment through the regulation of neuro-endocrine-immune network. However, the precise mechanism is still unclear. Here we want to emphasize that CSFP belongs to a new pharmacological experimental method, which is preliminary to solve the problems in vitro experiments of CHM for treating ND and needed further to clarify its action mechanisms.

**Figure 2:**
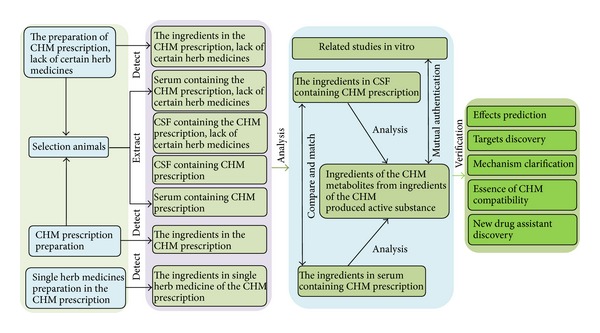
CSFP approaches for CHM research. It analyzes the ingredients of CHM, metabolites from ingredients of the CHM, and produced active substance in CSF containing CHM. Finally, it carries out experimental verifications to mutual authentication for the discovery of CHM-derived targets, effect prediction, mechanism clarification, and new drug assistant discovery using CSFP approach.

CSFP has become a helpful tool in understanding the fine details of drug-target interactions, efficacy, and mechanism of drugs. However, the CSFP still has some limitations. Firstly, whether the donor animals to extract CSF containing drugs are necessary for modeling, the difference of herbal ingredients in CSF between normal animal and model animal are still an unresolved issue. Secondly, in view of the complexity of CSF ingredients, more studies are required for better understanding the mechanism whereby the CSFP works. Finally, how to make an effective of these active ingredients in CHM, which could not passed through the blood-brain barrier, is still a puzzling question. Using CSFP to investigate CHM pharmacological effects and drug targets, attention should be paid to verify the results of CSFP analysis and mutual authentication. A diagram is proposed to exhibit the research approach of CSFP for CHM research (Figure 2). This approach is a combination of serum pharmacology, which comprises the core values for reflecting the ingredients, metabolites, and active substance correlated with CHM. The appropriate cellular models are also conducive to evaluate the effectiveness of CHM, which could be used to verify the results of CSFP analysis and mutual authentication. By integrating the chemical predictor, target predictor, and mutual authentication, a system of CSFP in CHM research was constructed. It systematically revealed the potential mechanisms of TCM. CSFP could be helpful to confirm the effective ingredients and promote drug discovery of CHM.

In summary, CSFP, as an improved pharmacology method to explore the material basis and action mechanism in vitro, not only breaks the theory that pharmacokinetics studies are limited to the overall function, but also offers new ideas for the selection of the appropriate cells in vitro culture system to study material basis and efficacy mechanism of CHM for treating ND. Moreover, the advancements in CSFP undoubtedly bring about a conceptual move in active ingredients discovery of CHM, make an operational shift in CHM research, and make a significant contribution to CHM modernization and globalization.

## Figures and Tables

**Table 1:** Brief introduction of mainly experimental techniques in CSFP.

Technique	Application fields	Advantage	Literatures
Percutaneous puncture	The acquisition of CSF containing drugs in rabbit and rat	Effectively avoid injuring the medulla oblongata, and blood vessel, significantly increase the rate of success in drawing CSF	[49]

Foramen magnum puncture	The acquisition of CSF containing drugs in SD rats	More visual and convenient to operate, causing less injury and complications and possessesing high rate of success	[50, 51]

Using microinjector via spinal dura mater puncture under direct vision	The acquisition of CSF containing drugs in SD rats	A fast, convenient, and clean technique for collecting CSF, increase the collection volume of CSF	[52]

The optimization to acquisition time of CSF containing drugs	The acquisition of CSF containing drugs in SD rats	Obtain optimal CSF containing drug	[43, 47, 48]

Analysis on transitional composition of drug in cerebrospinal fluid	Clarify main effective constituents	Convenient to clarify the true active ingredients	[69, 70]

Analysis on transitional composition association of drug in cerebrospinal fluid	Verify constituents or drug association and perfect quality standard	Simplify the complexity of constituents or drug and find out potential association	[75]
